# Marital status independently predicts pancreatic cancer survival in patients treated with surgical resection: an analysis of the SEER database

**DOI:** 10.18632/oncotarget.8467

**Published:** 2016-03-29

**Authors:** Xiao-Dong Wang, Jian-Jun Qian, Dou-Sheng Bai, Zhen-Nan Li, Guo-Qing Jiang, Jie Yao

**Affiliations:** ^1^ Department of Hepatobiliary and Pancreatic Surgery, Clinical Medical College of Yangzhou University, Subei People's Hospital of Jiangsu Province, Yangzhou, People's Republic of China

**Keywords:** pancreatic cancer, marital status, SEER, survival analysis

## Abstract

Marital status is an independent prognostic factor for survival in several cancers. To determine if that is also true for pancreatic cancer after surgical treatment, we examined 13,370 cases of pancreatic cancer reported to the Surveillance, Epidemiology, and End Results (SEER) database between 1988 and 2012. We found that patients who were widowed at the time of diagnosis were more likely to be female, a high percentage were elderly, a high ratio were diagnosed in early years, and a high proportion of tumors were located at the head of the pancreas (*P* < 0.05). Marital status was confirmed to be an independent prognostic factor in both univariate and multivariate analyses (*P* < 0.05). In those with localized disease, 5-year pancreatic cancer cause-specific survival was 6.5% lower in widowed patients than married ones (38.6% *vs*. 32.1%), though this difference was not significant in a multivariate analysis (*P* = 0.084). In those with regional disease or distant metastasis, univariate and multivariate analyses indicated marital status to be an independent prognostic factor (*P* < 0.05). Thus marital status is an important prognostic factor in pancreatic cancer, and widowed patients are at greater risk of death than others.

## INTRODUCTION

Pancreatic cancer is a devastating disease that remains the fourth leading cause of cancer-associated death in the United States [1, 2]. Despite advances in multi-modality therapy, pancreatic cancer remains extraordinarily lethal, with a 5-year overall survival rate of approximately 5% [1, 3]. There are, however, differences in patient survival related to the tumor's histology and its stage at diagnosis. Moreover, socioeconomic and demographic variables also likely play a role in the survival of pancreatic cancer patients, as has been demonstrated with other malignancies.

It is now recognized that marital status is a meaningful determinant of disease stage and grade at presentation, as well as a determinant of disease evolution after treatment. In colorectal cancer, for example, married individuals present with less advanced stage at diagnosis and exhibit better survival than unmarried individuals [4, 5]. Similarly, a larger population-based study of data from the Surveillance, Epidemiology and End Results (SEER) database indicates that for ten leading causes of cancer-related death, unmarried patients are at significantly greater risk of presentation with metastatic cancer, undertreatment, and death resulting from their cancer [6]. Although the impact of marital status on pancreatic cancer survival has not been extensively studied, the available data suggest marital status is an independent prognostic factor for both perioperative and long-term survival in patients with pancreatic cancer [7]. In that study, however, only a relative small number of patients were treated surgically, and unmarried individuals were not differentiated based on whether they were single, divorced and widowed. Therefore, to further investigate the relationship between marital status and pancreatic cancer outcomes, as well as the potential underlying mechanisms, we used data from the SEER cancer registry to explore the impact of marital status on pancreatic cancer cause-specific survival (PCSS) in patients after surgical resection.

## RESULTS

### Patient characteristics

There were 13,370 eligible cases (6,761 males and 6,609 females) of pancreatic adenocarcinoma reported in the SEER database from 1988 to 2012. Of those, 8,650 (64.70%) were married, 1,765 (13.20%) were widowed, 1,564 (11.70%) were single, and 1391 (10.40%) were divorced/separated. The median follow-up time was 22 months. The characteristics of patients with different marital statuses are summarized in Table 1. Significant (*P* < 0.001) parameters include the following. Patients who were widowed at the time of diagnosis were more frequently female and a high percentage were elderly. In addition, a high ratio were diagnosed in early years, and a high proportion of the tumors were located at the head of the pancreas.

**Table 1:** Baseline demographic and tumor characteristics of patients in SEER database

	Total	Married	Divorced/Separated	Single	Widowed	*P* value
Characteristic	(*n* = 13370)	(*n* = 8650)	(*n* = 1391)	(*n* = 1564)	(*n* = 1765)	
		*N* (%)	*N* (%)	*N* (%)	*N* (%)	
Sex						<0.001
Male	6761	5036(58.2)	574(41.3)	827(52.9)	324(18.4)	
Female	6609	3614(41.8)	817(58.7)	737(47.1)	1441(81.6)	
Age						<0.001
≦60	4388	2874(33.2)	583(41.9)	802(51.3)	129(7.3)	
>60	8982	5776(66.8)	808(58.1)	762(48.7)	1636(92.7)	
Race						<0.001
White	10883	7247(83.8)	1076(77.4)	1115(71.3)	1445(81.9)	
Black	1289	569(6.6)	217(15.6)	331(21.2)	172(9.7)	
Other^*^	1169	816(9.4)	95(6.8)	113(7.2)	145(8.2)	
Unknown	29	18(0.2)	3(0.2)	5(0.3)	3(0.2)	
Year of diagnosis						
1988-1996	2347	1504(17.4)	226(16.2)	238(15.2)	379(21.5)	
1997-2005	5684	3698(42.8)	565(40.6)	659(42.1)	762(43.2)	
2006-2012	5339	3448(39.9)	600(43.1)	667(42.6)	624(35.4)	
Primarysite location						
Head	9724	6272(72.5)	1021(73.4)	1164(74.4)	1267(71.8)	
Body	724	492(5.7)	62(4.5)	73(4.7)	97(5.5)	
Tail	1163	759(8.8)	123(8.8)	119(7.6)	162(9.2)	
Overlapping	604	408(4.7)	67(4.8)	62(4.0)	67(3.8)	
Unspecific	1155	719(8.3)	118(8.5)	146(9.3)	172(9.7)	
Pathological grading						0.054
I /II	7213	4675(54.0)	742(53.3)	829(53.0)	967(54.8)	
III/ IV	4139	2722(31.5)	439(31.6)	471(30.1)	507(28.7)	
Unknown	2018	1253(14.5)	210(15.1)	264(16.9)	291(16.5)	
Tumor Size(cm)						<0.001
≤4	8374	5453(63.0)	887(63.8)	953(60.9)	1081(61.2)	
>4	3081	2093(23.6)	294(21.1)	375(24.0)	373(21.1)	
Unknown	1915	1158(13.4)	210(15.1)	236(15.1)	311(17.6)	
SEER stage						0.172
Localized	1624	1040(12.0)	158(11.4)	204(13.0)	222(12.6)	
Regional	9135	5941(68.7)	979(70.4)	1047(66.9)	1168(66.2)	
Distant	2611	1669(19.3)	254(18.3)	313(20.0)	375(21.2)	

* Other includes American Indian/Alaska native, Asian/Pacific Islander, etc.

### Effect of marital status on PCSS

**Figure 1:**
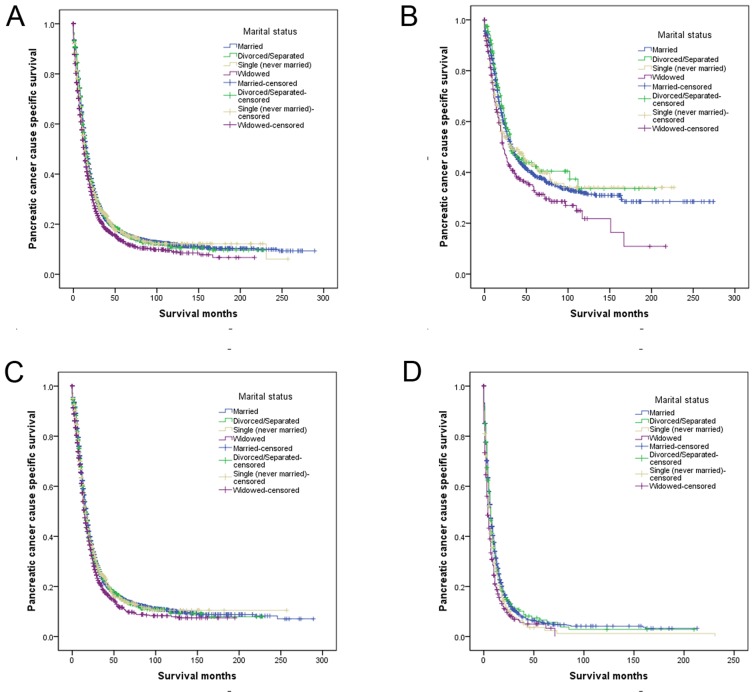
Survival curves in gastric patients according to marital status **a.** All stage; χ^2^ = 38.536, *P* < 0.001; **b.** Localized: χ^2^ = 9.572, *P*= 0.023; **c.** Regional: χ^2^ = 15.939, *P* =0.001; **d.** Distant: χ^2^ = 30.700, *P* < 0.001.

Patients in the SEER database who were widowed at the time of diagnosis had poorer PCSS than patients who were single or divorced/separated for the first 5 years following diagnosis (Figure 1A). Married patients had an increased risk of pancreatic cancer-caused mortality (hazard ratio [HR] 1.140; confidence interval [95% CI] 1.073-1.211), even after controlling for age at diagnosis, year of diagnosis, race/ethnicity, disease stage, and tumor type (Table 2).

**Table 2:** Univariate and multivariate survival analysis for evaluating the influence of marital status on pancreatic cancer cause-specific survival in SEER database

		Univariate analysis		Multivariate analysis	
Variable	5-year CCS	Log rank *χ*^2^ test	*P*	HR(95%CI)	*P*
**Sex**		1.924	0.165		NI
Male	15.4%				
Female	16.5%				
**Age**		72.907	<0.001		<0.001
≦60	18.4%			Reference	
>60	14.7%			1.262(1.208-1.318)	
**Race**		9.218	0.010		0.157
White	15.9%			Reference	
Black	14.7%			1.069(0.98-1.145)	0.055
Other^*^	18.2%			1.00(0.931-1.074)	0.999
**Year of diagnosis**		557.499	<0.001		<0.001
1988-1996	10.6%			Reference	
1997-2005	13.5%			0.834(0.791-0.879)	<0.001
2006-2012	22.2%			0.563(0.530-0.597)	<0.001
**Primary site location**		68.513	<0.001		0.001
Head	15.9%			Reference	
Body	19.0%			0.965(0.880-1.058)	0.448
Tail	18.5%			0.902(0.837-0.973)	0.008
Overlapping	12.1%			1.150(1.046-1.264)	0.004
Unspecific	14.1%			1.009(0.938-1.085)	0.805
**Grade**		300.500	<0.001		<0.001
I / II	19.0%			Reference	
III/ IV	10.9%			1.402(1.341-1.465)	<0.001
Unknown	15.0%			1.156 (1.089-1.227)	<0.001
**Tumor Size(cm)**		160.754	<0.001		<0.001
≤4	18.5%			Reference	
>4	13.8%			1.227(1.167-1.290)	<0.001
Unknown	8.3%			1.437(1.352-1.528)	<0.001
**SEER Stage**		3309.783	<0.001		<0.001
Localized	38.6%			Reference	
Regional	14.7%			1.890 (1.760-2.029)	<0.001
Distant	5.1%			3.803(3.509-4.121)	<0.001
**Marital Status**		38.536	<0.001		<0.001
Married	16.4%			Reference	
Divorced/Separated	16.3%			1.035(0.968-1.107)	0.312
Never married	16.6%			1.112(1.042-1.186)	0.001
Widowed	13.1%			1.140(1.073-1.211)	<0.001

* Other includes American Indian/Alaska native, Asian/Pacific Islander, and unknown.

NI: not included in the multivariate survival analysis.

Several other covariates were also predictive of mortality following surgical resection of pancreatic cancer. Older patients had poorer PCSS (HR 1.262; 95% CI 1.208-1.318), while white patients had a lower risk of mortality than black patients (HR 1.069; 95%CI 0.98-1.145). Patients diagnosed more recently experienced lower rates of pancreatic cancer-caused mortality (1997-2005, HR 0.834, 95%CI 0.791-0.879; 2006-2012, HR 0.563, 95%CI 0.530-0.597). Unsurprisingly, presenting with poorer grade, larger tumor or advanced stage were highly predictive of cancer-specific mortality (*P* < 0.05) (Table 2).

### Subgroup analysis of the effect of marital status

We next assessed of the effects of marital status on survival at each tumor stage. Among patients diagnosed with localized disease, 5-year PCSS was 6.5% lower for widowed than married patients (38.6% vs. 32.1%), though this difference was not significant in a multivariate analysis (*P* = 0.084). For patients with regional disease or distant metastasis, marital status was an independent prognostic factor associated with survival in univariate and multivariate analyses (*P* < 0.05). On the other hand, there was no apparent difference between the divorced/separated and married patients at any stage (Table 3, Figure 1B-1D).

**Table 3:** Univariate and multivariate analysis of marital status on pancreatic cancer cause specific survival based on different cancer stage

		Univariate analysis			Multivariate analysis	
Variable	5-year CCS	Log rank *χ*^2^ test	*P*	HR(95%CI)	*P*
**SEER Stage**					
**Localized**					
**Marital status**		9.572	0.023		
Married	38.6%			Reference	
Divorced/Separated	42.3%			1.048(0.819-1.340)	0.710
Never married	43.0%			1.174(0.948-1.452)	0.141
Widowed	32.1%			1.180(0.978-1.425)	0.084
**Regional**					
**Marital status**		15.939	0.001		
Married	15.3%			Reference	
Divorced/separated	14.8%			1.050(0.970-1.137)	0.229
Never married	14.7%			1.060(0.979-1.148)	0.149
Widowed	11.6%			1.117(1.038-1.203)	0.003
**Distant**					
**Marital status**		30.700	<0.001		
Married	5.3%			Reference	
Divorced/separated	6.6%			1.004(0.868-1.161)	0.961
Never married	3.6%			1.218(1.066-1.392)	0.004
Widowed	3.3%			1.135(1.002-1.286)	0.046

*P*-values refer to comparisons between two groups and were adjusted for primary site location, age, race, year of diagnosis, pathological grading, and tumor size as covariates.

NI: not included in the multivariate survival analysis.

## DISCUSSION

Married persons enjoy better overall health and a longer life expectancy than unmarried ones [8–10]. Research also indicates there is a survival advantage for married persons living with a chronic disease such as cancer. Indeed, marital status is an independent prognostic factor associated with survival in several cancers [4–6, 11–14]. In the present study, we used the SEER database to address this issue in the context of pancreatic cancer. We found that widowed patients had significantly poorer PCSS than their married counterparts. Moreover, the disadvantage to widowed patients persisted, even after adjusting for age, race, tumor location, grade and stage in multivariable analyses.

In an earlier study, Baine et al. showed that marital status is an independent prognostic factor associated with both perioperative and long-term survival in patients with pancreatic cancer [7]. However, they treated unmarried patients as single group and also did not distinguish patients based on disease stage. Our study indicates that unmarried patients are in fact a heterogeneous group, and that widowed patients have poorer survival outcomes than other unmarried patients. However, when considering localized pancreatic cancer, the number of patients in the widowed group was small, which can make the effect of marital status on PCSS difficult to detect and/or to quantify. This appears to have been the case in our study, as marital status emerged as a statistically significant factor in univariate analyses, but not in multivariate models.

One hypothesis to explain the less favorable prognosis in unmarried individuals is delayed diagnosis with advanced tumor stage. In our study group, however, the percentages of patients with localized and regional tumors or distant metastasis were comparable among the four subgroups. And clearly delayed diagnosis cannot explain the poorer survival outcomes in widowed patients treated with surgical resection. More likely, the relationship between marital status and survival is explained by psychosocial factors that are independent of tumor characteristics and the extent of treatment. Psychologically, a cancer diagnosis can be more distressing than other diagnoses [15]. Patients who are married display less distress, depression, and anxiety than their unmarried counterparts, as a partner can share the emotional burden and provide appropriate social support [16]. Marital status may also affect adherence to medical recommendations, leading to better compliance with treatment, delivery of treatment at more highly recognized centers, and acceptance of more aggressive treatment, all of which may result in better cancer control [17]. In addition, DiMatteo et al. observed a strong relationship between depression and non-adherence, and married patients displayed a lower risk of major depression [18]. Consistent with those ideas, when women with depression are diagnosed with breast cancer, they undergo definitive treatment less often and show poorer survival [19].

There is also evidence that a lack of psychosocial support and psychological stress alters immune function and contributes to tumor progression and mortality [20–22]. This may be made manifest by lower levels of natural killer cell activation, which may in turn lead to failure of cancer control [23]. Physicians should consider screening for depression among unmarried patients with cancer and refer patients to mental health specialists if symptoms are identified [6]. A widowed patient's loss of social support or their inability to cope with stress may lead to excess mortality [4, 24].

This study adds to current knowledge by answering in-depth questions about the relation between marital status and pancreatic cancer prognosis. However, it has several potential limitations. First, the marital status of a few patients did not stay the same, which could affect the results. Some patients classified as never married may have been cohabitating, while some patients classified as married may have separated or actually divorced. Second, the quality of the marriage can also impact the survival of pancreatic cancer patients. Marital distress has long term immune consequences and increases the risk of a variety of health problems [25]. Third, and perhaps most important, the SEER pancreatic cancer database lacks quality data on the surgery and systemic therapy. For example, the number of cycles or the chemotherapeutic regimen cannot be determined from the available data.

Despite these potential limitations, our results show that unmarried patients are a heterogeneous group, and that widowed patients are at a higher risk of death from their cancer than other patients. Psychosocial factors may be the primary reasons for the poorer survival outcomes in widowed patients. Physicians caring for these patients should be aware of their situation and provide closer care and interventions to help reduce their mortality risk.

## MATERIALS AND METHODS

The SEER database was utilized to access processed publically available data in 18 registries acquired from 1988 to 2012. The demographic and incidence data collected by the SEER registries cover approximately 28 percent of the US population, which are considered to be representative of the US population as a whole. The database includes sensitive patient information and has been widely used for studies of the relationship between marital status and survival outcomes of patients with cancer [6, 11, 13, 14, 26, 27].

ICD-O-3 (International Classification of Diseases for Oncology, 3^rd^ edition) morphology codes 8000, 8010, 8140, 8240, 8246, 8255, 8260, 8481, 8480, 8500 and 8574 were used with SEER*Stat software version 8.1.5 to identify pancreatic cancer. The inclusion criteria were as follows: (a) known marital status; (b) had a single primary pancreatic cancer or had more than one primary cancer but the pancreatic cancer was the first; (c) age at diagnosis was older than 18 years; (d) received surgical resection; (e) the cause of death and the number of months survived were both known. All pancreatic subsites, including C25.0-Head of pancreas, C25.1-Body of pancreas, and C25.2-Tail of pancreas, C25.8-Overlapping lesion of pancreas, C25.3-Pancreatic duct and unspecific location information were included in the study.

### Statistical analysis

Factors including marital status, age, gender, race, tumor location, extent of disease and year of diagnosis were evaluated. Race was divided into white, black and others. According to the SEER staging system, tumors that remain in situ or confined to the organ or origin were regarded as localized. Those that locally invaded or metastasized to regional lymph nodes were considered to be regional, while those that traveled to distant organs were categorized as distant. Within the SEER database, the marital status of the patient was recorded at the time of diagnosis. Marital status was coded as married, divorced, widowed, separated, never married and unmarried or domestic partner. Individuals in the separated and divorced groups were clustered together as divorced/separated [4, 14], while never married and unmarried or domestic partner were grouped together.

Differences in the distribution of covariates according to marital status were assessed using two-sided χ2 tests. Differences in survival were assessed using two-sided Kaplan-Meier log-rank tests. Multivariable Cox regression models were built for analysis of risk factors for survival outcomes. The primary endpoint of this study was PCSS, which was calculated from the date of diagnosis to the date of cancer-specific death. Deaths attributed to pancreatic cancer were treated as events, while deaths from other causes were treated as censored observations. All analyses were performed using the statistical software package SPSS for Windows, version 17 (SPSS Inc., Chicago, IL, USA). Statistically significance was set at two-sided *P* < 0.05.
